# High susceptibility to the novel antimicrobial zoliflodacin among *Neisseria gonorrhoeae* isolates in eight WHO Enhanced Gonococcal Antimicrobial Surveillance Programme countries in three WHO regions, 2021-2024

**DOI:** 10.1016/j.ijregi.2025.100624

**Published:** 2025-03-11

**Authors:** Susanne Jacobsson, Thitima Cherdtrakulkiat, Daniel Golparian, Lon Say Heng, Irving Hoffman, Manuel C. Jamoralin, Francis Kakooza, Rossaphorn Kittiyaowamarn, Peter Kyambadde, Pham Thi Lan, Venessa Maseko, Mitch Matoga, Etienne Müller, Thuy Thi Phan Nguyen, Vichea Ouk, Daniel Schröder, Vivi Setiawaty, Sonia B. Sia, Verawati Sulaiman, Mot Virak, Nguyen Thi Thuy Van, Ismael Maatouk, Teodora Wi, Magnus Unemo, WHO EGASP zoliflodacin study group

**Affiliations:** 1 Department of Laboratory Medicine, Faculty of Medicine and Health, WHO Collaborating Centre for Gonorrhoea and other Sexually Transmitted Infections, National Reference Laboratory for Sexually Transmitted Infections, Örebro University, Örebro, Sweden; 2 Division of AIDS and STIs, Department of Disease Control and Prevention, Bangrak STIs Center, Thailand Ministry of Public Health, Bangkok, Thailand; 3 National Center for HIV/AIDS, Dermatology and Sexually Transmitted Diseases, Phnom Penh, Cambodia; 4 UNC Project Malawi, Lilongwe, Malawi; 5 Research Institute for Tropical Medicine, Department of Health, Manila, Philippines; 6 Infectious Diseases Institute, Makerere University College of Health Sciences, Kampala, Uganda; 7 Sexually Transmitted Infections Program, Ministry of Health, Kampala, Uganda; 8 National Hospital of Dermatology and Venereology and Hanoi Medical University, Hanoi, Vietnam; 9 Centre for HIV and STIs, National Institute for Communicable Diseases, National Health Laboratory Service, Johannesburg, South Africa; 10 Ho Chi Minh City Hospital of Dermatology and Venereology, Ho Chi Minh City, Vietnam; 11 Laboratory of the National Institute of Public Health, Phnom Penh, Cambodia; 12 Sulianti Saroso Infectious Disease Hospital, Jakarta, Indonesia; 13 WHO Country Office, Hanoi, Vietnam; 14 Global HIV, Hepatitis and STI Programmes, WHO, Geneva, Switzerland; 15 Institute for Global Health, University College London (UCL), London, United Kingdom

**Keywords:** *In vitro* susceptibility, Zoliflodacin, WHO EGASP, *Neisseria gonorrhoeae*, Gonorrhea, Treatment

## Abstract

**Objectives:**

Zoliflodacin, a novel spiropyrimidinetrione, showed non-inferiority compared with recommended ceftriaxone plus azithromycin treatment in a recent global phase III randomized controlled trial for gonorrhea treatment. We evaluated the susceptibility of zoliflodacin among 2993 contemporary gonococcal isolates collected in 2021-2024 in eight World Health Organization (WHO) Enhanced Gonococcal Antimicrobial Surveillance Programme countries in the WHO Southeast Asian Region (Indonesia, Thailand), WHO Western Pacific Region (Cambodia, the Philippines, Viet Nam), and WHO African Region (Malawi, South Africa, Uganda).

**Methods:**

Minimum inhibitory concentrations (MICs) of zoliflodacin were determined using the agar dilution technique, and the zoliflodacin target gene (*gyrB*) was examined with Illumina sequencing.

**Results:**

Zoliflodacin exhibited high activity: MICs ranging from 0.001 to 1 mg/l and a modal MIC of 0.032 mg/l. The zoliflodacin MIC distribution showed mostly a wild-type profile; however, two isolates from Cambodia had MICs of 0.5 mg/l and 1 mg/l. These isolates also harbored the GyrB D429N mutation, associated with increased zoliflodacin MICs.

**Conclusions:**

We show a high susceptibility to zoliflodacin internationally, including against ceftriaxone- and azithromycin-resistant gonococcal strains. Our findings support the continued clinical development of zoliflodacin as a treatment for gonorrhea, although cautious and monitored introduction and continuous international resistance surveillance are imperative.

## Highlights

• Resistance in *Neisseria gonorrhoeae* has emerged to the last treatment, ceftriaxone.
• We show a high susceptibility to zoliflodacin among gonococci from three World Health Organization regions.
• We found two gonococcal isolates from Cambodia with increased zoliflodacin minimum inhibitory concentrations.
• Our study supports the further development of zoliflodacin for gonorrhea treatment.

## Introduction

The global prevalence of gonorrhea remains alarmingly high, as highlighted by the World Health Organization (WHO) (https://www.who.int/publications/i/item/9789240027077), and the treatment of gonorrhea is increasingly compromised because of the development or acquisition of antimicrobial resistance in *Neisseria gonorrhoeae* [[1], [2], [3]]. Over the recent decade, resistance to the last effective options for empirical treatment of gonorrhea, ceftriaxone and azithromycin, has emerged and spread worldwide [[2], [3], [4], [5], [6], [7], [8], [9]]. Of particular concern is the international dissemination of ceftriaxone-resistant, mosaic *penA-60* gonococcal strains since 2015, which now are common in countries such as Cambodia, China, and Viet Nam [3,4,[6], [7], [8], [9], [10], [11], [12], [13], [14]]. Some of these strains are extensively drug-resistant and resistant to ceftriaxone and azithromycin, including high-level resistance [7,[10], [11], [12], [13], [14]]. Given the potential for gonorrhea to become untreatable, there is an urgent need for new treatment options, as emphasized by the WHO and other public health agencies over the past decade [[15], [16], [17]].

A promising new antimicrobial is zoliflodacin, a first-in-class spiropyrimidinetrione that inhibits DNA gyrase (GyrB) using a new mechanism of action. A recent global phase III randomized controlled trial (RCT) for the treatment of uncomplicated gonorrhea in men and women demonstrated that a single 3 g oral dose of zoliflodacin was non-inferior to the recommended treatment of ceftriaxone (500 mg intramuscularly) plus azithromycin (1 g orally). Briefly, the global phase III RCT met its primary efficacy end point, with zoliflodacin demonstrating non-inferiority to ceftriaxone plus azithromycin in the treatment of urogenital gonorrhea. In the microbiological intent-to-treat population, zoliflodacin achieved a microbiological cure rate of 90.9%, a 5.3% difference compared with ceftriaxone plus azithromycin (96.2% cure rate). The non-inferiority of zoliflodacin was demonstrated within the pre-specified margin of 12% and within the margin of 10% as specified in US Food and Drug Administration guidance. Furthermore, microbiological cure rates at rectal and pharyngeal sites were comparable between treatment arms (secondary efficacy endpoints). Oral zoliflodacin 3 g was generally well-tolerated, and emergent adverse events were comparable between treatment arms, with no deaths or other serious adverse events reported (https://innovivainc.gcs-web.com/news-releases/news-release-details/innoviva-specialty-therapeutics-positive-phase-3-oral*,* https://www.clinicaltrials.gov/study/NCT03959527). Zoliflodacin has also shown high *in vitro* activity against *N. gonorrhoeae*, including extensively drug-resistant strains, with no clinical isolates exhibiting minimum inhibitory concentrations (MICs) beyond the wild-type range found in recent global gonococcal populations [18,19]. However, in laboratory experiments, mutants with increased zoliflodacin MICs, containing amino acid alterations in GyrB D429 or K450, have been selected [[20], [21], [22], [23], [24]]. A recent global whole genome sequencing (WGS)–based *in silico* mining study of 27,151 *N. gonorrhoeae* genomes from 1928 to 2021 identified only one isolate with a substitution in GyrB D429 (D429V) and no isolates with any GyrB K450 alteration [25]. Another mutation, GyrB S467N, has been identified as contributing to increased zoliflodacin MICs as a second-step mutation and first-step mutation (predisposing strains to develop higher MICs of zoliflodacin) [20,23]. The previously mentioned WGS-based *in silico* mining study found that 0.12% of *N. gonorrhoeae* genomes carried the GyrB S467N alteration [25].

To monitor global gonococcal antimicrobial resistance, the WHO developed the Global Gonococcal Antimicrobial Surveillance Programme [2,26,27]. In recent years, the WHO has improved standardization, quality assurance, and data collection through the WHO Enhanced Global Gonococcal Antimicrobial Surveillance Programme (EGASP), which includes globally representative sentinel countries. In 2023, WHO EGASP sentinel countries included Cambodia [7,10], Indonesia, Malawi, the Philippines, South Africa, Thailand [28], Uganda, Viet Nam [8], and Zimbabwe. In 2024, Argentina, Brazil, Côte d'Ivoire, India, and Qatar were in various stages of WHO EGASP implementation.

The objective of the present study was to report up-to-date *in vitro* zoliflodacin susceptibility data for clinical *N. gonorrhoeae* isolates (n = 2993) collected from 2021 to 2024 in eight WHO EGASP countries in the WHO Southeast Asian Region (Indonesia and Thailand), WHO Western Pacific Region (Cambodia, the Philippines, and Viet Nam), and WHO African Region (Malawi, South Africa, and Uganda). These data support further clinical development, registration, and introduction of zoliflodacin in clinical practice for gonorrhea treatment.

## Methods

### N gonorrhoeae isolates and antimicrobial susceptibility testing

A total of 2993 clinical *N. gonorrhoeae* isolates collected from 2021 to 2024 through the WHO EGASP were included. The isolates were cultured from men with urethral discharge in Cambodia (n = 482), Indonesia (n = 101), Malawi (n = 121), the Philippines (n = 843), South Africa (n = 597), Thailand (n = 250), Uganda (n = 350), and Viet Nam (n = 249).

All isolates were shipped frozen to the WHO Collaborating Centre for Gonorrhoea and Other STIs in Örebro, Sweden, in accordance with Material Transfer Agreements with three of the countries, and subsequently cultured on GCAGP agar medium (3.6% Difco GC Medium Base agar [BD Diagnostics, Sparks, MD, USA] supplemented with 1% hemoglobin [BD Diagnostics], 1% IsoVitalex [BD Diagnostics], and 10% horse serum) for 20-24 hours in a humid CO_2_-enriched atmosphere at 36 ± 1°C. The MICs (mg/l) of zoliflodacin (Entasis Therapeutics, Waltham, MA, USA) were determined by agar dilution according to Clinical and Laboratory Standards Institute guidelines (M07-A9 and M100-S24; www.clsi.org) on GCVIT agar plates (3.6% Difco GC Medium Base agar [BD Diagnostics] supplemented with 1% IsoVitalex [BD Diagnostics]). The WHO *N. gonorrhoeae* reference strains A, F, P [29], and ATCC 49226 were used for quality control. Mutations in the *gyrB* gene, with focus on the GyrB codons D429, K450, and S467, were identified using WGS, as previously described [5]. Full details of the WGS of WHO EGASP isolates will be presented elsewhere, i.e. when analyses have been finalized.

## Results

The results of the zoliflodacin susceptibility testing of the 2993 clinical *N. gonorrhoeae* isolates obtained from 2021 to 2024 in eight WHO EGASP countries in the WHO Southeast Asian Region, Western Pacific Region, and African Region are summarized in Table 1.

**Table 1:** Susceptibility to zoliflodacin in clinical *Neisseria gonorrhoeae* isolates (n = 2993) collected from 2021 to 2024 in eight WHO EGASP countries.

Country	Year of isolation	No. of isolates	Modal MIC (mg/l)	MIC range (mg/l)	MIC_50_ (mg/l)	MIC_90_ (mg/l)
Cambodia	2021	12	0.064	0.016-0.064	0.064	0.064
	2022	146	0.064	0.002-1	0.064	0.064
	2023	256	0.064	0.002-0.125	0.032	0.064
	2024	68	0.064	0.002-0.064	0.032	0.064
Indonesia	2023	101	0.016	0004-0.064	0.032	0.064
Malawi	2023	121	0.064	0.002-0.125	0.032	0.064
The Philippines	2022	255	0.016	0.002-0.125	0.032	0.064
	2023	588	0.032	0.002-0.125	0.032	0.064
South Africa	2022	303	0.032	0.002-0.25	0.032	0.064
	2023	294	0.064	0.001-0.125	0.032	0.064
Thailand	2022	100	0.032	0.016-0.125	0.032	0.125
	2023	150	0.064	0.002-0.25	0.032	0.064
Uganda	2022	141	0.032	0.002-0.064	0.032	0.064
	2023	209	0.016	0.002-0.064	0.016	0.032
Viet Nam	2023	249	0.064	0.002-0.25	0.064	0.125
Total		2993	0.032	0.001-1	0.032	0.064

EGASP, Enhanced Gonococcal Antimicrobial Susceptibility Programme; MIC, minimum inhibitory concentration; MIC_50_, concentration where 50% of isolates inhibited; MIC_90_, concentration where 90% of isolates inhibited; No., number; WHO, World Health Organization.

The MIC values for zoliflodacin ranged from 0.001 mg/l (one isolate from South Africa) to 1 mg/l (one isolate from Cambodia). The modal (most common) MIC among all isolates was 0.032 mg/l, varying from 0.016 mg/l (isolates from the Philippines in 2022 and from Indonesia and Uganda in 2023) to 0.064 mg/l (isolates from Cambodia in 2021-2024 and from Malawi, South Africa, Thailand, and Viet Nam in 2023). The overall MIC_50_ for all isolates was also 0.032 mg/l, but the MIC_50_ varied from 0.016 mg/l for isolates from Uganda in 2023 to 0.064 mg/l for isolates from Cambodia in 2021 and 2022 and Viet Nam in 2023. Finally, the overall MIC_90_ for all isolates was 0.064 mg/l, ranging from 0.032 mg/l for isolates from Uganda in 2023 to 0.125 mg/l for isolates from Thailand in 2022 and Viet Nam in 2023 (Table 1).

**Figure 1:**
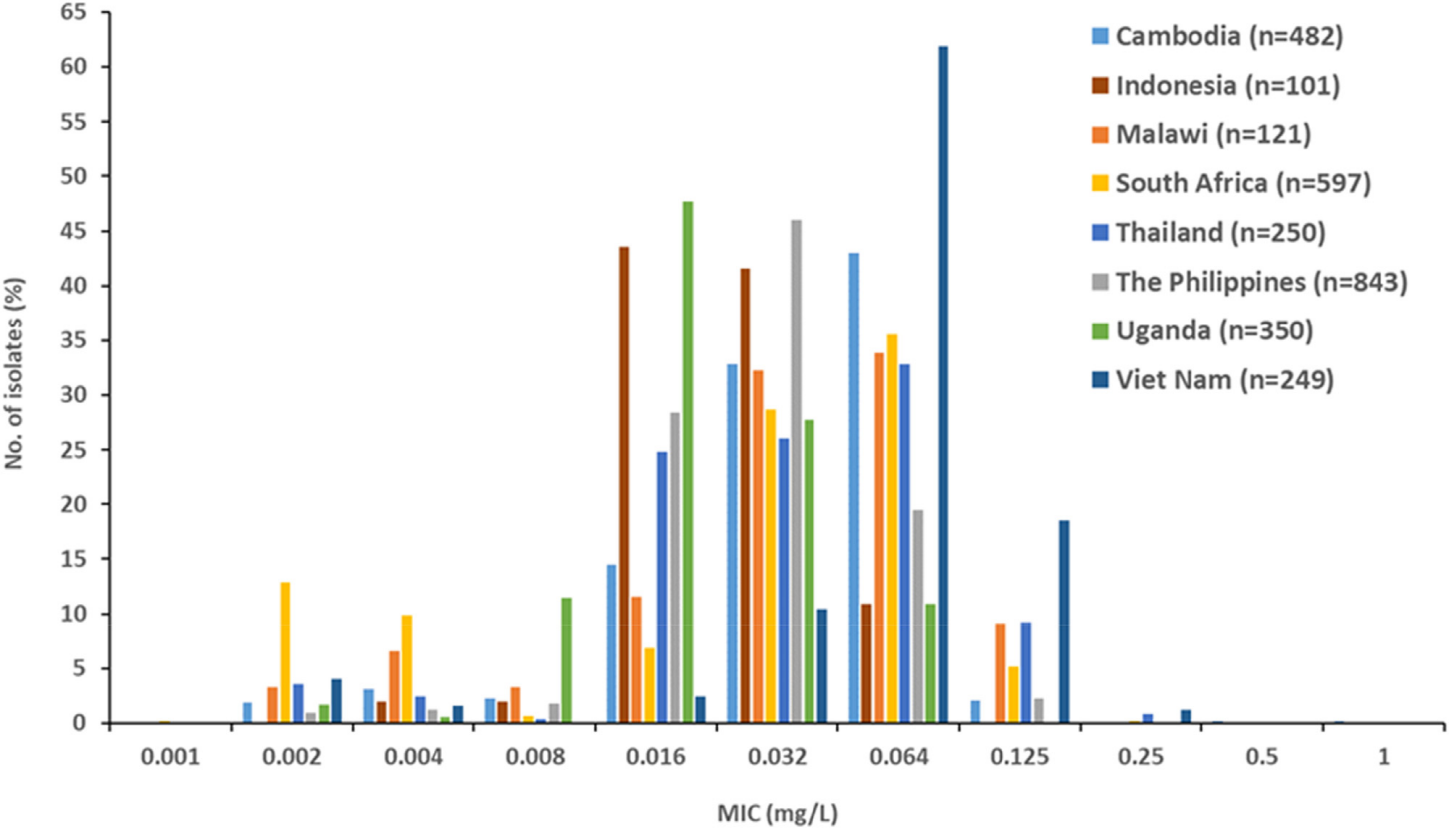
Zoliflodacin MIC distribution for WHO EGASP *Neisseria gonorrhoeae* isolates obtained in 2021 (n = 12), 2022 (n = 945), 2023 (n = 1968), and 2024 (n = 68). EGASP, Enhanced Gonococcal Antimicrobial Susceptibility Programme; MIC, minimum inhibitory concentration; WHO, World Health Organization.

The zoliflodacin MIC distribution for all the WHO EGASP isolates from 2021 to 2024 (n = 2993) is shown in Figure 1. Briefly, the WHO EGASP isolates mainly represented a zoliflodacin wild-type MIC distribution. However, two isolates cultured in Cambodia in 2022 had MICs of 0.5 mg/l and 1 mg/l (all other isolates had a zoliflodacin MIC of ≤0.25 mg/l), and the MIC distribution for isolates from Viet Nam was slightly shifted toward higher MICs than the isolates from other countries (Figure 1).

The two isolates from Cambodia with the highest recorded MICs in the study (0.5 and 1 mg/l) harbored the GyrB D429N mutation, which has been associated with increased zoliflodacin MICs [[20], [21], [22], [23], [24]]. However, no amino acid alterations were found in GyrB K450. The GyrB S467N mutation [20,23,25] was found in 14 isolates (0.5%; Viet Nam [n = 10], the Philippines [n = 2], Cambodia [n = 1], and Thailand [n = 1]).

## Discussion

In the present international WHO EGASP study, zoliflodacin, the first-in-class spiropyrimidinetrione that recently demonstrated non-inferiority to the currently recommended ceftriaxone-azithromycin dual therapy for uncomplicated gonorrhea in a global phase III RCT, was shown to exhibit a high *in vitro* susceptibility against a contemporary international collection of *N. gonorrhoeae* isolates. The collection comprised 2993 gonococcal isolates collected from 2021 to 2024 in eight globally representative sentinel countries included in the WHO EGASP and included many isolates with resistance to ceftriaxone (4%), cefixime (9%), and azithromycin (4%) (https://www.who.int/westernpacific/publications/i/item/9789240102927) [7,8,10]. The zoliflodacin MIC_90_ for the isolates with resistance to ceftriaxone, cefixime, and azithromycin was the same as for isolates susceptible to these antimicrobials, i.e. 0.064 mg/l. All these results are encouraging and align with previously performed zoliflodacin susceptibility studies examining *N. gonorrhoeae* isolates from Europe, South Africa, the USA, Thailand, and China, as well as a study including a global collection of international reference strains and isolates selected for diverse antimicrobial resistance phenotypes, all of which similarly showed high *in vitro* susceptibility to zoliflodacin [18,19,30,31].

However, the present study also, for the first time, identified the GyrB D429N mutation in clinical *N. gonorrhoeae* isolates, i.e. two isolates from Cambodia collected in 2022, both showing MICs of 0.5 and 1 mg/l for zoliflodacin. In previous zoliflodacin *in vitro* susceptibility studies and genomic studies, only one zoliflodacin-resistant isolate (MIC = 8 mg/l) has been reported. This was an *N. gonorrhoeae* isolate cultured in Japan in 2000, harboring the zoliflodacin resistance-associated GyrB D429V mutation. However, this strain or the zoliflodacin resistance determinant did not appear to have been further transmitted [25].

The limitations of the present study are the inherent limitations in the WHO EGASP and include the limited number of participating sentinel countries and gonococcal isolates examined and inclusion of only urethral discharge specimens from males. However, to address these limitations, the WHO EGASP has expanded from one country in 2015 (Thailand) to nine countries, representing three WHO regions in 2023. The number of intra-country sentinel clinical sites are also increasing nearly annually. In addition, Argentina, Brazil, Côte D'Ivoire, India, and Qatar are currently in different stages of EGASP implementation. Finally, the WHO EGASP has published supplementary protocols, including sampling of anorectal and oropharyngeal specimens, and these protocols are currently implemented in some of the WHO EGASP countries.

In conclusion, the high *in vitro* susceptibility among contemporary *N. gonorrhoeae* isolates (n = 2993) collected in eight WHO EGASP countries in the WHO Southeast Asian Region (Indonesia and Thailand), WHO Western Pacific Region (Cambodia, the Philippines, and Viet Nam), and WHO African Region (Malawi, South Africa, and Uganda) during 2021-2024, together with the promising results in the recent global zoliflodacin phase III RCT for the treatment of uncomplicated gonorrhea, supports further clinical development, registration, and introduction of zoliflodacin for the treatment of uncomplicated gonorrhea. However, strict consideration regarding several key questions for the optimal introduction of zoliflodacin in the treatment of uncomplicated urogenital and extragenital gonorrhea in males and females [32] and, subsequent to the introduction, rigorous and ongoing monitoring of zoliflodacin susceptibility (MIC- and genome-based) will be essential to promptly identify and address any emergence or transmission of resistance.

## Declarations of competing interest

The authors have no competing interests to declare.
